# Knowledge translation in surgery: a scoping review of implementation strategies, effectiveness and contextual barriers and enablers

**DOI:** 10.1186/s12913-025-13369-2

**Published:** 2025-12-05

**Authors:** Elizabeth Manafò, Elyette Lugo, Amit Jain, Lisa Petermann, Benjamin Davies, Olesja Hazenbiller, Janneke I. Loomans, Muzahem M. Taha, Klaus John Schnake, Michael P. Kelly, Asdrubal Falavigna, Anne Versteeg, Richard Bransford, Riccardo Cecchinato, Charles Fisher, AO Knowledge Translation Steering Committee

**Affiliations:** 1 EXEP Consulting Inc, Calgary, AB Canada; 2 John Hopkins School of Medicine, Baltimore, MD United States of America; 3 University of Cambridge, Cambridge, UK; 4 AO Foundation, Davos Platz, Switzerland; 5 Orthopaedic Surgery Department, Kirkuk University, Kirkuk, Iraq; 6 Center for Spinal and Scoliosis Surgery, Malteser Waldkrankenhaus St. Marien Erlangen, Erlangen, Germany; 7 Department of Orthopedics and Traumatology, Paracelsus Private Medical University, Nuremberg, Germany; 8 Department of Orthopaedic Surgery, University of California, San Diego, CA United States of America; 9 University of Caxias do Sul, Caxias do Sul, Brazil; 10 Department of Orthopaedic Surgery, University of Toronto, Toronto, ON Canada; 11 Department of Orthopaedic Surgery, University of Washington, Harborview Medical Center, Seattle, WA United States of America; 12 Department of Biomedical Sciences for Health, University of Milan, Milan, Italy; 13 IRCCS Galeazzi-Sant’Ambrogio Hospital, Milan, Italy; 14 Department of Orthopaedics, Division of Spine, University of British Columbia and Vancouver General Hospital, Vancouver, BC Canada

**Keywords:** Knowledge translation, Implementation science, Surgeons, Innovation, Practice change

## Abstract

**Background:**

Knowledge translation (KT) interventions are essential for implementing evidence-based practices in healthcare. However, despite their proven effectiveness in addressing global health challenges, KT strategies in surgery remain challenging to apply. This scoping review examines KT strategies in surgery, their effectiveness, and key barriers and enablers to their implementation.

**Methods:**

This scoping review followed the Arksey and O’Malley and Levac et al. frameworks, integrating the RE-AIM (Reach, Effectiveness, Adoption, Implementation, Maintenance) and the PRISM (Practical Robust Implementation Sustainability Model) models to evaluate the effectiveness of knowledge translation interventions in surgical practice change and associated contextual barriers and facilitators. A systematic search was conducted across MEDLINE (PubMed, OVID), CINAHL (EBSCO), and PsycINFO (ProQuest). Articles were screened using predefined selection criteria, emphasizing experimental and quasi-experimental studies. Data extraction categorized KT interventions: knowledge diffusion, dissemination, and implementation approaches.

**Results:**

A total of 34 studies met the inclusion criteria. Most were hospital-based (88%) and focused on guideline adherence. The review identified three primary KT strategies: (i) educational materials and educational outreach, (ii) reminders and prompts, and (iii) audit and feedback systems. The most effective KT strategies used a combination of these interventions to maximize impact. Barriers included physician resistance, limited leadership support, financial constraints, and workflow disruptions, while enablers included institutional leadership, structured training programs, financial incentives, and interdisciplinary collaboration. A notable finding was the lack of standardized validation processes for adopting changes in the surgical setting, which often burdens individual surgeons and their institutions, thereby constraining both capacity and motivation for practice change.

**Conclusions:**

Findings suggest that layered, interdisciplinary KT strategies are the most effective for driving surgical practice change and overcoming institutional barriers. The integrated application of RE-AIM and PRISM frameworks proved valuable in assessing the interventions’ sustainability and real-world effectiveness. This comprehensive analysis contributes to the growing body of knowledge on effective implementation strategies in surgical settings and provides a foundation for future practice improvement initiatives. Future research should focus on refining KT methodologies, expanding implementation frameworks, and addressing barriers to sustainability across diverse surgical settings.

**Supplementary Information:**

The online version contains supplementary material available at 10.1186/s12913-025-13369-2.

## Background

Implementing evidence-based practice in healthcare is essential for incorporating scientific knowledge into healthcare systems, practices and policies to improve health outcomes [1]. Knowledge translation (KT) interventions, more broadly referred to as implementation science, has gained global recognition as a structured approach to expanding and disseminating research within complex healthcare systems [2]. Despite the substantial volume of research, their application remains relatively new within clinical settings, particularly within the surgical setting [3]. Surgical interventions have inherent characteristics - such as challenges with standardization, blinding, and patient recruitment in research studies - that make it difficult to apply conventional evidence-based medicine standards that work well for other medical treatments [4].

Surgical practice presents unique opportunities for implementation science due to its direct and measurable impact on patient outcomes. Integrating evidence-based interventions in surgery can also drive improvements in cost-effectiveness, surgical technique, technology, professional development and quality improvement [1, 4]. KT aims to drive high-value, high-quality surgical care that benefits both patients and healthcare providers while also ensuring relevance and responsiveness to practice, policy changes and cost. However, translating research findings into surgical practice is particularly challenging due to the variability in adoption, regional/country practice differences, the lack of standardized processes for validating/approving innovative techniques and knowledge, and the inherent need for interdisciplinary collaboration [5, 6]. The inconsistent uptake of new evidence in surgical practice and its extent of implementation suggests that significant barriers or facilitators exist, necessitating formalized implementation science KT frameworks to support and sustain evidence-based practice change [1, 7].

Arroyo et al. (2021) conducted a scoping review exploring factors that promote surgeon practice change, offering valuable insights into barriers and enablers to the adoption of evidence-based practices in surgical settings [4]. However, their review, while highly informative, lacks a specific focus on distinct KT and implementation science intervention types, particularly in assessing the effectiveness of these strategies in surgical practice. This limitation underscores the need for an updated review that provides a more detailed examination of specific KT interventions and their impact on promoting practice change in surgery.

This scoping review aims to identify the essential characteristics of effective KT interventions that facilitate sustainable surgical practice change and inform policy development. This study will summarize the existing literature on KT strategies in surgery, assess their effectiveness, and explore barriers and enablers to their implementation. By applying an implementation science perspective, this review seeks to enhance the adoption of evidence-based innovations in surgical practice, ultimately improving patient outcomes and advancing the quality of surgical care.

## Methods

This scoping review followed the Arksey and O’Malley [8] and Levac et al. [9] frameworks to examine KT surgical interventions. This approach maps relevant research on implementation science in surgical settings, identifying key themes and summarizing the literature to generate research questions and inform practice recommendations.

To enhance the usability and applicability of the findings, two implementation science frameworks: RE-AIM (Reach, Effectiveness, Adoption, Implementation, Maintenance) which focuses on the tactical approaches and PRISM (Practical Robust Implementation Sustainability Model) which focuses on the contextual factors [10] were applied within the Arksey and O’Malley framework. These frameworks were specifically used to guide the formulation of research questions and data analysis to ensure a structured evaluation of both intervention effectiveness (tactics) and contextual factors influencing implementation (barriers and enablers) (Table 1).

**Table 1:** Implementation science Framework - Informing scoping review research question

Evaluation Approach	Research Question
RE-AIM (tactical assessment – Reach, Effectiveness, Adoption, Implementation and Monitoring)	1. What interventions (i.e., tactical approaches) or frameworks exist to facilitate change in surgical practice outcomes (effectiveness)?
PRISM (contextual factors – Implementation & Sustainability, External Environment, multi-level perspectives at the individual, organizational and system level)	2. Within these interventions, what are the barriers and enablers (i.e., context) to surgical practice change and the implementation of new surgical innovations?

### Search strategy

The search was conducted across three databases: MEDLINE (PubMed, OVID), CINAHL (EBSCO), and PsycINFO (ProQuest). A preliminary literature scan identified keywords, which were refined and validated through discussions with surgeons and KT specialists. The search strategy focused on terms related to surgeons, surgical procedures, implementation science, guideline adherence, behavior change, and barriers/enablers to KT adoption. Detailed search terms are listed in S1 Terms, and an example search strategy is provided in S2.

### Selection process

The selection process followed a two-stage screening approach. One investigator (EM) conducted the initial screening, applying the inclusion criteria (Table 2) and screening article titles and abstracts using *Rayyan* screening software. This software facilitates the independent review and assessment of articles for inclusion. Relevant details were extracted, including study design, population, intervention, and outcomes.

In the second stage, two investigators (EM and EL) independently reviewed the initial yield, applying the exclusion criteria (Table 2). Investigators used their KT (EM) and surgical care (EL) expertise to screen and filter results, enhancing the scoping review’s focus on both aspects. Articles that received unanimous agreement proceeded to full-text review. Disagreements were discussed, and a joint decision was made regarding inclusion. Additional senior investigators (AJ: surgical expertise and LP: KT expertise) were available to ensure academic rigor when needed.

**Table 2:** Final scoping review article selection criteria

Inclusion
• Topic focusses on review questions related to effectiveness, barriers and enablers in surgery/surgical behavior/by surgeons • Peer-reviewed journal articles • Experimental and quasi experimental, including systematic reviews of studies with an experimental and quasi experimental design • Verification: Published within last 10 years (2014-current) • Verification: Published in a peer-reviewed journal • Verification: Articles published in English
Exclusion
• Articles that do not address practice change/intervention • Articles that only discuss Clinical Practice Guidelines or intervention implementation without addressing the knowledge translation strategies used to facilitate change • Articles that are not inclusive of surgery (medical specialty) • Focused on medical and resident training (educational focus)*

*KT interventions that directly change clinical practice and patient care, rather than educational interventions that primarily target knowledge acquisition or skill development

### Data extraction and synthesis

One investigator (EM) developed a data extraction table in *Microsoft Excel* as an initial review of findings to reassess the article selection criteria. Utilizing *Adobe Acrobat AI Assistant*, the investigator first extracted data on country of origin, study summary, population, innovation, and key facilitators and barriers (e.g., attitudes, knowledge, benefits, limitations).

The research team (BD, CF, OH, JL, AV) reviewed preliminary findings and selection criteria, ensuring the study design aligned with the scoping review methodology. Studies were analyzed based on key themes, grouping interventions by type and identifying major barriers and enablers.

The final selection emphasized experimental designs, such as randomized controlled trials (RCTs), quasi-experimental studies, and systematic reviews of these study types, as they provide more substantial evidence for causal relationships between KT interventions and practice change. This emphasis is particularly important in surgical contexts where patient safety and clinical outcomes depend on the successful implementation of practices. In addition, previous studies exploring KT in surgical settings focused almost exclusively on contextual factors, thereby providing a novel opportunity for this research to contribute to the growing space in implementation science.

### Final selection and secondary search

One investigator (EL) manually extracted data from the primary yield and finalized the data extraction table in *Microsoft Excel*. Both investigators (EM and EL) independently reassessed the extracted data to ensure consistency with the final selection criteria. A secondary search (EM) was conducted using citations from selected articles, utilizing PubMed’s Similar Articles & Cited By features, and hand-searching reference lists for additional relevant studies.

The following variables were included in the final table: Source citation, Country of publication, Article type, Brief summary, Population(s), Study setting, Surgical procedure(s), Description of KT intervention/tactics/elements, Outcome, Barriers, and Enablers. KT intervention/tactics/elements were organized based on their KT intervention tactic: knowledge diffusion, knowledge dissemination, and knowledge implementation. Knowledge diffusion communicates information passively with minimal customization, such as publications, general guidelines or large-scale presentations. Knowledge dissemination tailors the method and message to a specific audience and/or context. Knowledge implementation further adapts this information to actively facilitate practice changes on the regional and local level.

## Results

### Selection of sources of evidence

The systematic search identified 970 articles from databases and an additional 10 from a rapid initial search. After removing 131 duplicates and one retracted article, one reviewer screened 848 records by title and abstract. A total of 630 articles were excluded for being outside the study scope, leaving 218 articles for further screening based on selection criteria by two reviewers. Of these, 143 were further excluded, and 76 full-text articles were assessed for eligibility by both reviewers.

Exclusions were made based on focus, outcomes, or intervention relevance. The reviewers reached 80% agreement in their evaluations, leading to the inclusion of 67 articles (88%) and flagging nine articles (12%) for further review. Articles were flagged as uncertain if one reviewer marked them as “unsure” while the other marked “yes” or “no”. Those with an unsure/no combination were excluded (*n* = 5), while those with an unsure/yes combination were retained for additional review (*n* = 4). Articles were flagged as uncertain when they fell into gray areas of our inclusion criteria, such as studies with both educational and practice-change components (unclear primary focus), insufficient detail about KT strategies or outcomes, unclear relevance to surgical practice, or ambiguous distinction between barriers/enablers and intervention components. These uncertain cases were resolved through discussion between reviewers to ensure consistent application of inclusion criteria. Following the iterative process adopted by the working group, a secondary screening was conducted to limit the final yield to experimental and quasi-experimental studies and review articles that included experimental studies. This additional filtering removed 37 additional articles, bringing the total final selection to 34. A PRISMA-ScR diagram of the search process and output is included in S3.

### Characteristics of included studies

The majority of studies were published in the USA, UK, and Europe, with most articles published from 2020 onward. Most studies (88%, *n* = 30) were conducted in hospital settings, and more than half (65%, *n* = 22) involved interdisciplinary teams, rather than surgeons alone, emphasizing the collaborative nature of KT implementation in surgical practice (Table 3). The included articles covered a wide range of surgical procedures.

Most studies (62%, *n* = 21) focused on implementing guidelines. These interventions covered the entire surgical care course, from pre-op planning to post-operative care, focusing on general surgery, gastrointestinal procedures, and specialized protocols for pediatric and ICU care. Many studies assessed procedure-specific protocols across various surgical specialties, including orthopedic, cardiac, and urologic surgeries.

**Table 3:** Selected studies characteristics

Study Characteristic	*n*=	%
Geographic location		
*** USA***	**22**	**65%**
* Canada*	-	
* UK*	6	18%
* Europe*	-	
* Australia/New Zealand*	3	9%
* South America*	1	3%
* Nordic (Finland)*	1	3%
* X (Other)*	1	3%
Population		
* Surgeon*	12	35%
***Interdisciplinary (including surgeon)***	**22**	**65%**
Sub-specialties:		
• General (*n*=8, 67%)		
• Colorectal/Gastrointestinal (*n*=7, 58%)		
• Orthopedic (*n*=4, 33%)		
• Urological (*n*=3, 25%)		
• Head and neck (*n*=2, 17%)		
• Hand (*n*=2, 17%)		
• Cardiac/cardiovascular (*n*=2, 17%)		
• Pediatric (*n*=2, 17%,)		
• (Robotic – radical prostatectomy, transoral, pediatric and general (*n*=5))		
Setting		
*** Hospital***	**30**	**88%**
* Academic/Research centre*	-	
* Clinical centre*	1	3%
* Outpatient surgical centre*	2	6%
* Community*	1	3%
Article Type		
* Clinical trial*	2	6%
* Meta analysis*	-	
* RCT*	2	6%
* Review*	7	21%
* Systematic review*	10	29%
*** Quasi-experimental***	**13**	**38%**
Publication year		
*** 2020-2024***	**19**	**56%**
* 2014-2019*	15	44%
Surgical intervention		
* Surgical innovation*	13	38%
*** Implementation of guidelines/best practices***	**21**	**62%**

### Synthesis

The scoping review identified three primary KT interventions: (i) educational materials and educational outreach, (ii) reminders and prompts, and (iii) audit and feedback systems.

Educational materials and outreach aimed to provide clear guidance through various formats such as surgical technique videos, clinical decision support tools, standardized protocols, simulation training, wet/dry labs, and mentorship programs. These methods were associated with improvements in health care team communication, adherence to safety protocols, and surgical decision-making [11–16], particularly in multidisciplinary teams. Studies implementing these approaches reported enhanced interdisciplinary coordination which resulted in higher patient satisfaction [13, 17].

Reminders and prompts functioned as reinforcement tools, supporting sustained practice change. These interventions were delivered via email alerts, posters, dashboard reporting, and real-time monitoring of practice patterns [4, 13, 17–26]. The outcomes of these strategies varied. For example, Johnston et al. [27] reported high interrater reliability (κ = 0.713) for reminder tools and significant improvements in compliance with evidence-based surgical practices. However, variability remained in briefing compliance and team participation, demonstrating the challenges in ensuring uniform uptake of reminders across surgical teams.

Audit/feedback is a systematic monitoring approach used to assess compliance and drive practice improvements through data-driven feedback cycles. These methods included baseline audits, follow-up compliance checks, and specialized audit tools to monitor adherence to surgical protocols [11, 12, 15, 15, 19, 21, 25, 26, 28–31]. Mcgee et al. [22] found that superficial surgical site infections decreased by 30% when bundle implementation was monitored, with additional improvements in clinical metrics such as length of hospital stay, which decreased from 10.8 to 8.3 days. However, not all postop outcomes improved, as readmission rates increased from 0 to 20%. Studies consistently reported that higher adherence to bundled interventions correlated with better surgical outcomes [32], particularly in reducing surgical site infections when prevention measures were followed [33]. A summary of three priority KT approaches, their descriptions, and their associated outcomes in the practice change context is included in Table 4.

**Table 4:** Priority KT approach, description and outcomes

Priority approaches				
Approach/Intervention	Articles	Tactic Aim	Tactic Description	Tactic Outcome & Considerations
Educational materials and educational outreach				
Educational materials (including printed, online)	Ariyo et al., 2019; Arora et al., 2022; Colebatch & Lockwood, 2020; Davis et al., 2022; Feinberg et al., 2018; Gillepsie & Marshall, 2015; Hatharaliyadda et al., 2024; Howell & Hirth, 2023; Hull et al., 2017; Jabbour et al. 2018; Kalkwarf et al., 2024; Lagoo et al., 2019; Lawrie et al., 2022; Sivarajan et al., 2015; Smith et al., 2024; Tomsic et al., 2020; Tonutti et al., 2017; Vitous et al., 2023; Vohra et al., 2018	Aim: Provide clear, accessible guidance on new techniques, technologies, and care protocols.	Educational materials are deployed through three main approaches: (1) Surgical Technique Demonstration using videos of procedures, visual equipment demonstrations, and protocol documentation; (2) Clinical Decision Support through evidence summaries, standardized protocols, selection criteria, and implementation guides; (3) Patient Care Integration via team coordination protocols, preadmission guidelines, safety checklists, and outcome expectation materials. Together, these components form a comprehensive educational approach that provides visual, evidence-based, and procedural guidance to support surgical practice.	• Team Dynamics Improvements: o Better communication among healthcare teams o Enhanced care coordination in multidisciplinary contexts o Higher reported patient satisfaction • Clinical Protocol Adherence: o Improved adherence to safety protocols o Better compliance with evidence-based surgical practices o Enhanced surgical decision-making o Consistent guideline adherence • Implementation Value: o Creates knowledgeable team environment o Establishes standardized clinical practices o Serves as effective foundational tool for surgical innovations
Educational outreach	Aloia et al., 2019; Close et al., 2023; Cundy et al., 2014; Davis et al., 2022; Deftereos, 2022; Feinberg et al., 2018; Gillepsie & Marshall, 2015; Gomes et al., 2021; Hatharaliyadda et al., 2024; Howard et al., 2021; Howell & Hirth, 2023; Jabbour et al. 2018; Johnston et al., 2014; Kalkwarf et al., 2024; Lagoo et al., 2019; Lawrie te al., 2022; Ninan et al., 2023; Sivarajan et al., 2015; Smith et al., 2024; Tomsic et al., 2020; Tonutti et al., 2017; Treadwell et al., 2014; Vitous et al., 2023; Vohra et al., 2018	Aim: Deploy through multiple channels to enhance direct interaction and practical skill development, supported by experienced practitioners and leadership teams to improve team coordination and system-level operating room processes.	Educational outreach employs practical, hands-on approaches through four integrated strategies: (1) Surgical Skills Development using wet/dry labs, simulations, and practical demonstrations for specialized procedures; (2) Operating Room Team Integration through staff role training, robotic system usage, safety checklist implementation, and multidisciplinary collaboration; (3) Surgical Mentorship via expert guidance, clinical champion coaching, on-site facilitation, and peer learning; and (4) Surgical System Implementation support for transitions to new approaches, optimized scheduling, protocol implementation, and team adaptation to technologies. This comprehensive approach focuses on experiential learning and direct support from experienced practitioners to build technical competence and team coordination.	• Skill Proficiency: o Improved technical competence with new surgical techniques o Enhanced confidence in using advanced surgical technologies o Reduced learning curve for complex procedures • Team Performance: o Better role integration among multidisciplinary team members o Improved coordination during surgical procedures o More effective adaptation to workflow changes • Implementation Success: o Smoother transition to new surgical approaches o Higher adoption rates of innovative techniques o More consistent application of protocols o Reduced resistance to technological changes • System-Level Improvements: o More efficient operating room scheduling o Better resource utilization o Enhanced patient safety through standardized practices • Knowledge Transfer: o Sustainable skill development through mentorship o Creation of local expertise and champions o Ongoing support mechanisms beyond initial implementation
Reminders and prompts				
Reminders and prompts	Ariyo et al., 2019; Arora et al., 2022; Crosby et al., 2023; Deftereos et al., 2022; Howell & Hirth, 2023; Hull et al., 2017; McGee et al., 2019; Ninan et al., 2023; Sivarajan et al., 2015; Smith et al., 2024; Tomsic et al., 2020; Tonutti et al., 2017; Vohra et al., 2018	Function as continuous reinforcement tools integrated with other knowledge translation strategies to support practice change, incorporating email communication to ensure stakeholder awareness and engagement.	Reminders and prompts function as continuous reinforcement tools through two complementary approaches: (1) Types of Reminders including educational prompts that bridge knowledge-practice gaps, regular staff monitoring and feedback mechanisms, dashboard reporting systems, and real-time monitoring of practice patterns such as prescribing behaviors; and (2) Implementation Methods featuring email communications, visual poster reminders, structured feedback loops, systematic patient follow-up processes, and regular clinical audits with practitioner feedback. This tactical approach embeds ongoing reinforcement within clinical workflows to sustain behavior change and ensure consistent adherence to new surgical practices over time.	· Procedural Compliance Improvements: o Enhanced briefing compliance across surgical teams o Increased team participation in required protocols o Significant improvements in surgical count practices adherence · Clinical Outcome Benefits: o Reduced risk-adjusted superficial surgical site infection rates o Improved bundle adherence directly correlated with lower infection rates o Significant reductions in SSI rates with prevention measure compliance · Implementation Considerations: o Ongoing challenges with sustained protocol adherence despite reminders o Resource limitations identified as barrier to full implementation o Reinforcement needed to maintain practice changes over time · Effectiveness Factors: o Most effective when integrated with other knowledge translation strategies o Regular reminders shown to bridge gap between knowledge and practice o Simple interventions (like posters/emails) produced measurable practice changes o Structured follow-up mechanisms reinforced initial adoption of new practices
Audit and feedback				
Audit and feedback	Colebatch & Lockwood, 2020; Close et al., 2023; Crosby et al., 2023; Davis et al., 2022; Feinberg et al., 2018; Gillepsie et al., 2015; Gomes et al., 2021; Hull et al., 2017; Johnston et al., 2014; Kalkwrft et al., 2024; Tomsic et al., 2020; Treadwell et al., 2014; Vohra et al., 2018	Aim: Monitor implementation systematically, assess compliance, and drive practice improvements through data-driven feedback cycles in surgical settings.	Audit and feedback employs a data-driven approach through four interconnected components: 1) Audit Methods including baseline and follow-up audits, specialized tools like JBI, PACES and GRiP, custom assessment tools, and registry analytics; (2) Feedback Mechanisms with regular clinician feedback, dashboard reporting, real-time monitoring, structured feedback loops, and performance metrics; (3) Areas of Focus such as protocol compliance, briefing components, technology usage patterns, guideline adherence, and prescribing practices; and (4) Implementation Features incorporating regular monitoring cycles, quality improvement integration, multidisciplinary involvement, linking results to practice changes, and continuous evaluation.	· Measurable Clinical Improvements: o Reduced length of hospital stay o Decreased mortality trends o Significantly reduced surgical site infection rates o Increased appropriate surgical intervention rates · Enhanced Protocol Adherence: o Higher bundle adherence directly correlated with better clinical outcomes o Improved compliance with audit criteria across surgical teams o More consistent implementation of evidence-based practices o Majority of studies reported significant infection rate reductions with protocol adherence · Reduced Practice Variations: o Decreased variability in Enhanced Recovery Pathway implementation o More standardized care delivery post-implementation o Reduction in opioid use with reduced prescriber variability o More consistent application of clinical guidelines · Implementation Insights: o Identified specific areas requiring attention (e.g., readmission rates increased) o Provided data-driven justification for continuing successful practices o Created accountability mechanisms for maintaining practice changes o Enabled targeted refinements to implementation strategies

Peer-reviewed journals primarily provide evidence for knowledge translation in surgical settings, with their absence potentially linked to adverse outcomes from the early adoption of new techniques [34]. Meetings, webinars, and peer collaborations serve as interactive platforms to share evidence-based knowledge, teach specific surgical techniques, and implement standardized protocols through structured educational session [17, 18, 20, 22, 24–26, 33]. These collaborative approaches may enhance multidisciplinary team engagement, improve surgical communication, and aid in the implementation of complex procedures like robot-assisted surgery [35, 36].

Despite the benefits of KT interventions, several barriers hindered their implementation. Physician preference for existing practices [20], resistance to change, and limited managerial support [37] were commonly cited challenges. Departmental policies [16, 20], high technology costs, and resource limitations posed additional obstacles, particularly in smaller hospitals where financial constraints limited access to KT tools [12]. System integration issues and workflow disruptions further complicated implementation, while steep learning curves and limited training opportunities made it difficult for surgeons to adopt new practices [16, 36]. Insufficient compliance monitoring and inconsistent adherence to evaluation protocols created additional challenges in ensuring the long-term sustainability [16].

Overcoming these challenges requires a structured and comprehensive support system. Institutional leadership plays a critical role by providing clear guidelines and methodical planning to facilitate KT adoption [18, 22, 27, 33]. This is further supported by comprehensive education and training programs, such as simulation-based learning, mentorship, and team-based training, to improve the effectiveness of KT interventions by providing surgical teams with the skills required to implement new practices [22, 26]. Interdisciplinary collaboration networks and industry partnerships foster knowledge sharing and facilitate the adoption of innovative approaches [12, 13, 18, 29]. Evidence-based quality management strategies and technological support intersect to enable data-driven improvements and minimize learning curves through advanced visualization and guidance systems [3–5, 7]. Additionally, financial and organizational incentives also contribute to sustaining KT implementation by aligning quality improvement objectives with hospital reimbursement structures and resource allocation [38, 39]. A summary of the key barriers and enablers influencing KT implementation in surgical practice is provided in Table 5. The categories were developed based on a summary of key themes that emerged from our analysis to help organize and present the barriers and facilitators identified across the included studies. These categories represent the main areas where implementation factors clustered, providing a practical framework for understanding the range of challenges and supports encountered in surgical KT interventions.

**Table 5:** Barriers and enablers to KT in surgical practice change

Category	Implementation Factor | Conceptual Definition	Barriers	Facilitators
Leadership and organizational factors	Governance, management support, and institutional policies	Lack of managerial and institutional support, departmental policies limiting adoption, competitive dynamics between departments, poor infrastructure	Strong managerial and institutional leadership support, structured planning, leadership engagement, service-specific customization, engagement of clinical champions
Cultural and professional collaboration	Workplace culture, professional relationships, and interdisciplinary cooperation	Physician preference and resistance to change, cultural resistance, skepticism, lack of buy-in, inconsistent team engagement	Collaborative networks, knowledge-sharing platforms, multidisciplinary teamwork, cross-specialty platforms, peer recommendations
Education and training	Knowledge transfer and skill development	Limited training opportunities, insufficient awareness, limited documentation and training tools, surgeons’ unfamiliarity	Structured training programs, continuous education, tailored coaching and mentorship, team training initiatives, collaborative learning
Financial and resource management	Resource allocation: human, material, and financial considerations.	High implementation costs, limited resources for smaller hospitals, financial burdens, cost constraints, resource constraints for auditing	Financial incentives aligned with quality improvement, reimbursement structures, high institutional volume advantages, resource provision
Technical implementation and technology	Deploying and integrating new technologies, tools, or systems into existing workflows.	Complexity of integration, technical incompatibilities, system integration challenges, limited flexibility, poor coordination	Shorter learning curves through improved design, remote capabilities, robust pre-operative imaging, implementation science insights
Evidence and quality assurance	Methods to measure outcomes, ensure compliance, and maintain standards through data collection and analysis.	Lack of long-term impact data, insufficient compliance monitoring, inadequate adherence, variable adoption patterns	Evidence-based metrics, registry data and transparency, strong audit systems, structured feedback mechanisms, continuous monitoring

**Fig. 1:**
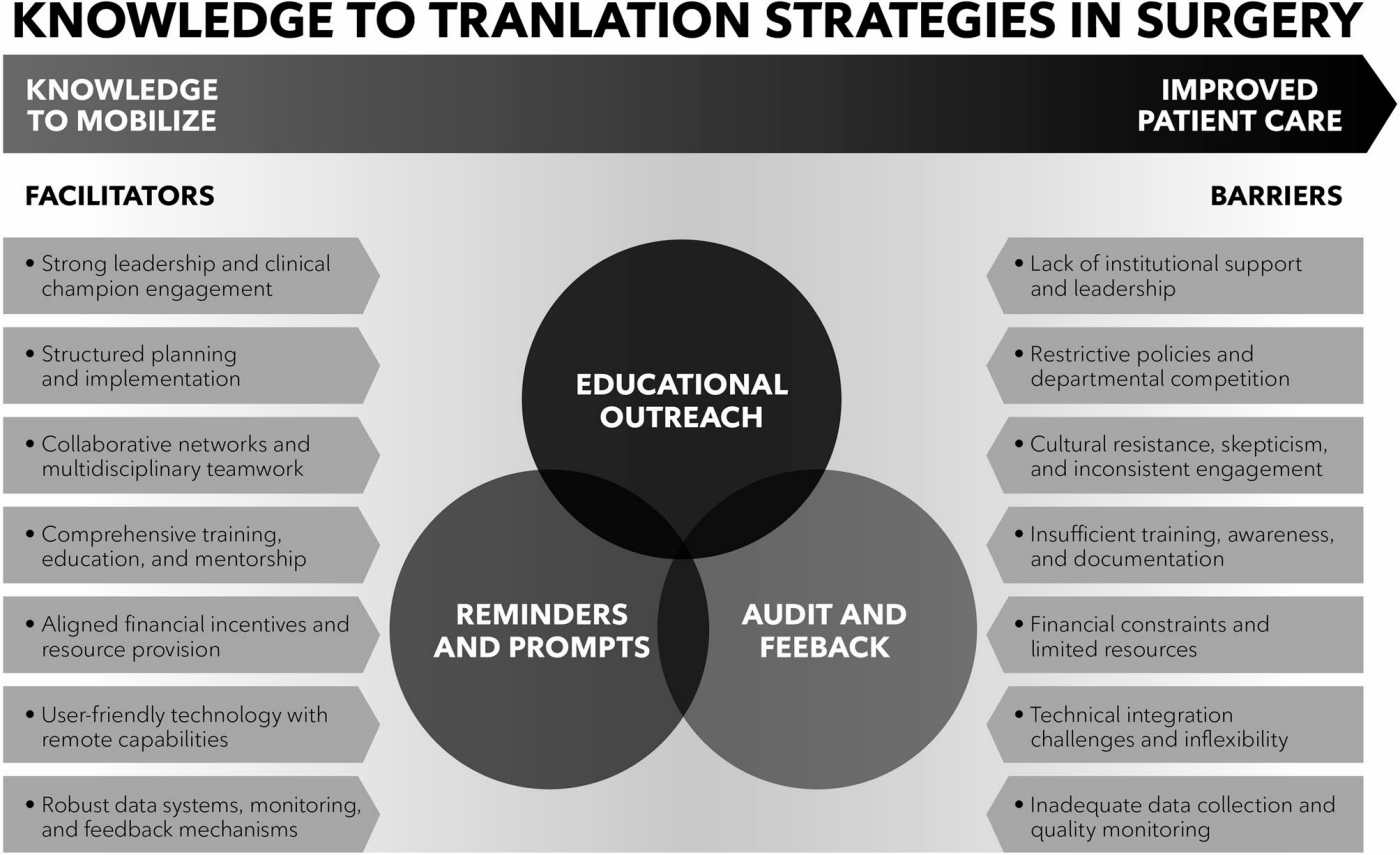
Knowledge translation tactics, enablers and barriers in surgical care

A summary of key findings on tactics, enablers and barriers is presented in Fig. 1.

## Discussion

This scoping review highlights the complexity, interdependence and need for additional research to implement knowledge translation (KT) interventions in surgical practice.

### Complexity of multidisciplinary approaches

This scoping review highlights the complexity in implementing knowledge translation (KT) interventions in surgical practice. The gap between how we expect surgical practice changes to occur (Work-as-Imagined, WAI) and how they unfold in real clinical settings (Work-as-Done, WAD) represents a fundamental challenge in KT implementation [40]. Understanding this gap is essential for effective implementation, as solutions should not force WAD to comply with WAI, but rather acknowledge these differences and find ways to bridge them. As such, sustainable surgical practice change depends on engagement beyond just surgeons, requiring a layered approach that integrates diverse stakeholders across institutional levels, where strong leadership and cultural receptivity are as essential as financial resources. Effective KT requires a multifaceted approach that integrates structured education, interdisciplinary collaboration, continuous monitoring, and strong institutional support. This comprehensive approach ensures that innovations are introduced and sustained in clinical practice, reflecting the broader trends in implementation science. The evolution of KT strategies in the surgical field reflects a shift from passive knowledge diffusion toward more integrated, multi-faceted implementation approaches. This shift recognizes that changing surgical practice involves more than just information dissemination. It requires addressing multiple barriers, such as institutional infrastructure, resource allocation, and interdisciplinary collaboration.

### Interdependence of strategies

Effective knowledge translation in surgical settings requires multiple concurrent approaches rather than standalone tactics, as evidenced by the superior results of bundled interventions that combine educational materials with real-time reinforcement, reminders, and structured feedback systems. Our findings suggest that the most effective KT interventions use multiple strategies simultaneously rather than relying on a single method. Educational materials combined with educational outreach remain foundational, but their impact is maximized when combined with real-time reinforcement through reminders, prompts, and audit and feedback systems [23, 28, 31, 41].

Regular monitoring and feedback are crucial for keeping quality improvements on track, ensuring that new practices continue to be followed, and making adjustments when needed to achieve the best results. Creating systematic ways to give and receive feedback helps healthcare teams adapt and stick with practice changes, which ultimately leads to better surgical procedures and patient care [28, 42]. Hatharaliyadda et al. [32] demonstrated that adherence to surgical site infection prevention protocols improved using dashboard reporting and continuous feedback loops, which minimized disruptions to surgeon workflow.

The interventions have shown the greatest effectiveness in improving adherence to best practices, particularly in surgical site infection prevention and safety protocols. Mcgee et al. [22] found that superficial surgical site infections decreased by 30% when bundle implementation was monitored, with additional improvements in length of hospital stay, which decreased from 10.8 to 8.3 days. However, the success of these interventions depends not only on the strategies themselves but also on the broader system in which they are implemented. Achieving meaningful and sustainable surgical practice change requires a layered, interdisciplinary approach that integrates multiple strategies and engages key stakeholders across institutional levels. While adequate financial resources are crucial in fostering quality improvement and innovation, they alone are insufficient alone. Successful implementation depends on strong and sustained institutional support alongside active engagement across interdisciplinary teams, extending beyond a surgeon-centric focus to include a broader network of healthcare professionals and perioperative stakeholders [12, 19, 21, 22, 33, 43].

### Gaps in current research

Based on the studies we reviewed, no research has comprehensively examined all these interconnected factors together in a single framework. Current research is also limited by potential publication bias that may not include enough examples of failed implementation attempts. The predominance of hospital-based studies in this review highlights the strong relationship between institutional infrastructure and the successful implementation of KT interventions, particularly in the face of resource barriers [12, 27, 44]. Our findings are limited by the predominance of studies from high-income countries in our sample, with most low- and middle-income countries (LMICs) being indirectly excluded through our search strategy and inclusion criteria. This geographic bias may have prevented us from capturing a greater variety of study settings and implementation contexts that exist in LMICs, where resource constraints, healthcare infrastructure, and cultural factors may present different barriers and facilitators for KT interventions in surgical practice. The lack of representation from diverse economic and healthcare contexts limits the generalizability of our findings and may have resulted in an incomplete understanding of the full spectrum of implementation challenges and strategies relevant to global surgical practice. Several studies identified cultural resistance, lack of leadership, and limited institutional support as significant barriers to KT into practice [23, 25, 36, 39]. These findings raise important questions about the scalability and equity of surgical innovation adoption, particularly in smaller hospitals or resource-limited settings [16, 23, 24, 44]. Strategies to address these disparities are essential to ensure that advancements in surgical practice benefit all patients, regardless of geographic or institutional constraints. A practical approach to overcoming these challenges is the adoption of gradual integration region specific strategies rather than large-scale, immediate changes.

Several limitations should be acknowledged to provide context for interpreting the findings of this review. The selection criteria focused on KT interventions that guide decision-making, which, while ensuring relevance, may have excluded other valuable perspectives. Exclusivity in study design, a 10-year publication time frame, language limitations to English, and database selection may have further limited the inclusion of relevant studies. To mitigate potential biases, multiple investigators conducted screening, selection, and data abstraction, supplemented by a secondary search strategy. However, despite these efforts, some relevant studies may not have been captured despite these efforts. Furthermore, the search strategy prioritized KT interventions, which may have introduced bias toward intervention-focused literature. The predominance of studies reporting successful KT interventions suggests potential publication bias, as unsuccessful or mixed-outcome studies may be underrepresented in the literature.

Most included studies were conducted in the United States, limiting the generalizability to other healthcare systems, particularly non-differentiation between urban and rural contexts. Additionally, the predominant focus on hospital-based settings, with limited representation of other surgical environments (such as outpatient facilities), may have limited the scope of the findings. The heterogeneity of study designs and outcomes makes it challenging to have direct comparisons in synthesizing this work to inform decision-making. However the cohesiveness of findings regardless of the diversity of surgical disciplines, environments and care pathways may suggest repeatable patterns and findings across studies. Additionally, the predominance of studies reporting successful KT interventions suggests a potential publication bias, meaning that unsuccessful implementation attempts may be underrepresented. These gaps highlight a need for more comprehensive research methodologies that can effectively capture the intricate interactions between various implementation strategies, diverse stakeholders, and different healthcare contexts to meaningfully advance changes in surgical practice.

## Conclusions

This study has contributed to the understanding of implementation science in surgery by demonstrating several key findings. Continuous monitoring and structured feedback are a crucial in refining surgical procedures and improving patient care. The findings emphasized the need for addressing institutional infrastructure, and resource allocation as key contextual factors to ensure successful adoption of new practices.

Gradual implementation, rather than immediate full-scale changes, better facilitate institutional support and resource management. Interdisciplinary collaboration remains essential in overcoming barriers such as cultural resistance and leadership challenges, reinforcing the need for system-wide engagement in surgical practice change.

Future research should validate these findings through key informant interviews in diverse surgical settings. Further data collection on barriers, facilitators, and best practices will also help refine KT strategies, enabling surgical teams to anticipate challenges and adapt approaches that support sustainable surgical innovation.

## Supplementary Information

Supplementary Material 1

## Data Availability

No datasets were generated or analysed during the current study.

## Abbreviations

KT: Knowledge translation
ScR: Scoping review

## References

[1] Nasser JS, Chung KC. Implementation science in surgery: translating outcomes to action. Plast Reconstr Surg. 2023;151(2):237–43. 36696301 10.1097/PRS.0000000000009822

[2] Chambers DA, Evans KM. Navigating the field of implementation science towards maturity: challenges and opportunities. Implement Sci. 2024;19(1):26. 38481286 10.1186/s13012-024-01352-0 PMC10936041

[3] Beauchemin M, Cohn E, Shelton RC. Implementation of clinical practice guidelines in the health care setting: a concept analysis. ANS Adv Nurs Sci. 2019;42:307–24. 30839334 10.1097/ANS.0000000000000263 PMC6717691

[4] Arroyo NA, Gessert T, Hitchcock M, Tao M, Smith CD, Greenberg C, et al. What promotes surgeon practice change?? A scoping review of innovation adoption in surgical practice. Ann Surg. 2021;273(3):474–82. 33055590 10.1097/SLA.0000000000004355 PMC10777662

[5] Jain M, Duh QY, Hirose R, Sosa JA, Suh I. A model for the institutional adoption of innovative surgical techniques. Surgery. 2020;168(2):238–43. 32376046 10.1016/j.surg.2020.03.018

[6] Byrnes A, Mudge A, Clark D. Implementation science approaches to enhance uptake of complex interventions in surgical settings. Aust Health Rev. 2020;44(2):310–2. 30982502 10.1071/AH18193

[7] Simunovic M, Urbach DR, Fahim C, et al. High-intensity vs low-intensity knowledge translation interventions for surgeons and their association with process and outcome measures among patients undergoing rectal cancer surgery. JAMA Netw Open. 2021;4(7):e2117536. 34269805 10.1001/jamanetworkopen.2021.17536 PMC8285735

[8] Arksey H, O’Malley L. Scoping studies: towards a methodological framework. Int J Social Res Methodology: Theory Pract. 2005;8(1):19–32.

[9] Levac D, Colquhoun H, O’Brien KK. Scoping studies: advancing the methodology. Implement Sci. 2010;5(69). 10.1186/1748-5908-5-69 PMC2954944 20854677

[10] Re-AIM.org. Available from: https://re-aim.org.

[11] Gillespie BM, Marshall A. Implementation of safety checklists in surgery: a realist synthesis of evidence. Implement Sci. 2015;10:137. 26415946 10.1186/s13012-015-0319-9 PMC4587654

[12] Colebatch E, Lockwood C. Enhanced perioperative nutritional care for patients undergoing elective colorectal surgery at calvary North Adelaide hospital: a best practice implementation project. JBI Evid Synth. 2020;18(1):1–19. 31290790 10.11124/JBISRIR-2017-003994

[13] Deftereos I, Hitch D, Butzkueven S, Carter V, Fetterplace K, Fox K, et al. Implementing a standardised perioperative nutrition care pathway in upper Gastrointestinal cancer surgery: a mixed-methods analysis of implementation using the consolidated framework for implementation research. BMC Health Serv Res. 2022;22(1):1–15. 35209897 10.1186/s12913-022-07466-9 PMC8876395

[14] Lagoo J, Singal R, Berry W, Gawande A, Lim C, Paibulsirijit S, et al. Development and feasibility testing of a device briefing tool and training to improve patient safety during introduction of new devices in operating rooms: best practices and lessons learned. J Surg Res. 2019;244:579–86. 31446322 10.1016/j.jss.2019.05.056

[15] Davis TL, Schäfer WLA, Blake SC, Close S, Balbale SN, Perry JE, Zarate RP, Ingram M, Strople J, Johnson JK, Holl JL, Raval M. V. A qualitative examination of barriers and facilitators of pediatric enhanced recovery protocol implementation among 18 pediatric surgery service. 2022;3(1):91. 10.1186/s43058-022-00329-8 PMC9389824 35982503

[16] Jabbour M, Newton AS, Johnson D, Curran JA. Defining barriers and enablers for clinical pathway implementation in complex clinical settings. Implement Sci. 2018;13(1):139. 30419942 10.1186/s13012-018-0832-8 PMC6233585

[17] Tonutti M, Elson DS, Yang GZ, Darzi AW, Sodergren MH. The role of technology in minimally invasive surgery: state of the art, recent developments and future directions. Postgrad Med J. 2017;93(1097):159–67. 27879411 10.1136/postgradmedj-2016-134311

[18] Arora A, Brunet A, Oikonomou G, Tornari C, Faulkner J, Jeyarajah J, et al. Establishing and integrating a transoral robotic surgery programme into routine oncological management of head and neck cancer – a UK perspective. J Laryngol Otol. 2022;136(12):1231–6. 35189991 10.1017/S002221512100476X

[19] Crosby HW, Pierce R, Regunath H. Implementation of a multidisciplinary team– based clinical care pathway is associated with increased surgery rates for infective endocarditis. J Clin Outcomes Manage. 2023;30(2):42–8.

[20] Howell JW, Hirth MJ. Around the global hand table: hand surgeon and therapist perspectives on overcoming barriers to relative motion orthotic intervention in the management of zones V-VI finger extensor tendon repairs. J Hand Ther. 2023;36(2):400–13. 37037729 10.1016/j.jht.2023.02.006

[21] Hull L, Athanasiou T, Russ S. Implementation science: a neglected opportunity to accelerate improvements in the safety and quality of surgical care. Ann Surg. 2017;265(6):1104–12. 27735828 10.1097/SLA.0000000000002013

[22] McGee MF, Kreutzer L, Quinn CM, Yang A, Shan Y, Halverson AL, et al. Leveraging a comprehensive program to implement a colorectal surgical site infection reduction bundle in a statewide quality improvement collaborative. Ann Surg. 2019;270(4):701–11. 31503066 10.1097/SLA.0000000000003524 PMC7775039

[23] Ninan A, Grubb LM, Brenner MJ, Pandian V. Effectiveness of interprofessional tracheostomy teams: a systematic review. J Clin Nurs. 2023;32(19):6967–86. 37395139 10.1111/jocn.16815

[24] Sivarajan G, Taksler GB, Walter D, Gross CP, Sosa RE, Makarov DV. The effect of the diffusion of the surgical robot on the Hospital-level utilization of partial nephrectomy. Med Care. 2015;53(1):71–8. 25494234 10.1097/MLR.0000000000000259 PMC4707949

[25] Tomsic I, Heinze NR, Chaberny IF, Krauth C, Schock B, von Lengerke T. Implementation interventions in preventing surgical site infections in abdominal surgery: a systematic review. BMC Health Serv Res. 2020;20(1):1–21. 10.1186/s12913-020-4995-z PMC7083020 32192505

[26] Vohra HA, Ahmed EM, Meyer A, Kempfert J. Knowledge transfer and quality control in minimally invasive aortic valve replacement. Eur J Cardiothorac Surg. 2018;53:ii9–13. 29718232 10.1093/ejcts/ezy077

[27] Johnston FM, Tergas AI, Bennett JL, et al. Measuring briefing and checklist compliance in surgery: a tool for quality improvement. Am J Med Qual. 2014;29(6):491–8. 24270170 10.1177/1062860613509402

[28] Feinberg J, Flynn L, Woodward M, Pennell C, Higham H, Morgan L, et al. Improving emergency surgical care for patients with right iliac fossa pain at a regional scale: a quality improvement study using the supported champions implementation strategy. Int J Surg. 2018;57:105–10. 30114495 10.1016/j.ijsu.2018.08.003

[29] Gomes ET, Galvão MCB, Shimoda GT, de Oliveira LB, de Araújo Püschel VA. Surgical counts in open abdominal and pelvic surgeries in a university hospital: a best practice implementation project. Int J Evid Based Healthc. 2021;19(1):84–93. 10.1097/XEB.0000000000000253 33570336

[30] Kalkwarf KJ, Bailey BJ, Wells A, Jenkins AK, Smith RR, Greer JW, et al. Using implementation science to decrease variation and high opioid administration in a surgical ICU. J Trauma Acute Care Surg. 2024;97(5):716–23. 38685205 10.1097/TA.0000000000004365 PMC11502286

[31] Treadwell JR, Lucas S, Tsou AY. Surgical checklists: a systematic review of impacts and implementation. BMJ Qual Saf. 2014;23(4):299–318. 23922403 10.1136/bmjqs-2012-001797 PMC3963558

[32] Hatharaliyadda B, Schmitz M, Mork A, Osman F, Heise C, Safdar N, et al. Surgical site infection prevention using strike teams: the experience of an academic colorectal surgical department. J Healthc Qual. 2024;46(1):22–30. 38166163 10.1097/JHQ.0000000000000412

[33] Ariyo P, Zayed B, Riese V, Anton B, Latif A, Kilpatrick C, et al. Implementation strategies to reduce surgical site infections: a systematic review. Infect Control Hosp Epidemiol. 2019;40(3):287–300. 30786946 10.1017/ice.2018.355

[34] Garas G, Cingolani I, Panzarasa P, Darzi A, Athanasiou T. Network analysis of surgical innovation: measuring value and the virality of diffusion in robotic surgery. PLoS One. 2017;12(8):e0183332. 28841648 10.1371/journal.pone.0183332 PMC5571947

[35] Chang SL, Kibel AS, Brooks JD, Chung BI. The impact of robotic surgery on the surgical management of prostate cancer in the USA. BJU Int. 2015;115(6):929–36. 24958338 10.1111/bju.12850

[36] Cundy TP, Marcus HJ, Hughes-Hallett A, Najmaldin AS, Yang GZ, Darzi A. International attitudes of early adopters to current and future robotic technologies in pediatric surgery. J Pediatr Surg. 2014;49(10):1522–6. 25280660 10.1016/j.jpedsurg.2014.05.017

[37] Lawrie L, Gillies K, Duncan E, Davies L, Beard D, Campbell MK. Barriers and enablers to the effective implementation of robotic assisted surgery. PLoS One. 2022;17(8):e0273696. 36037179 10.1371/journal.pone.0273696 PMC9423619

[38] Close S, Blake SC, Davis TT, Balbale SN, Perry JE, Weingard R, Ingram MC, Schäfer W, Strople J, Raval MV. Implementation of enhanced recovery protocols for gastrointestinal surgery in children: practical tools from key stakeholders. 2023;284:204–2012. 10.1016/j.jss.2022.11.071. 10.1016/j.jss.2022.11.071 PMC9911379 36586313

[39] Howard R, Delaney L, Kilbourne AM, Kidwell KM, Smith S, Englesbe M, et al. Development and implementation of preoperative optimization for high-risk patients with abdominal wall hernia. JAMA Netw Open. 2021;4(5):e216836. 33978723 10.1001/jamanetworkopen.2021.6836 PMC8116983

[40] Hollnagel E, Clay-Williams R, Vitous CA, Rivard SJ, Ervin JN, Duby A, Hendren S, Suwanabol PA. Work-as-imagined and work-as-done. In: Handbook of human factors and ergonomics. 5th Taylor Francis; Vitous CA, Rivard SJ, Ervin JN, Duby A, Hendren S, Suwanabol PA. Reducing ileostomy readmissions: using implementation science to evaluate the adoption of a quality improvement initiative. Dis Colon Rectum. 2023;66(12):1587–94. 37018541 10.1097/DCR.0000000000002684

[41] Smith CJ, Schäfer WLA, Wilberding MJ, Reiter A, Sullivan GA, Hu A, et al. Fidelity of enhanced recovery protocol implementation with assessment of hospital-specific materials. J Surg Res. 2024;302:469–75. 39167901 10.1016/j.jss.2024.07.087 PMC11490383

[42] Aloia TA, Keller DS, Kowalski RB, Lin H, Luciano MM, Myers JA, et al. Enhanced recovery program implementation: an evidence-based review of the art and the science. Surg Endosc. 2019;33(11):3833–41. 31451916 10.1007/s00464-019-07065-6

[43] A T, BMZ H. Innovation, development and clinical adoption of ureteroscopy: a time trend since its first inception. BJU Int. 2024;134(4):551–3. 39082311 10.1111/bju.16488

[44] Leggott KT, Martin M, Sklar D, Helitzer D, Rosett R, Crandall C, et al. Transformation of anesthesia for ambulatory orthopedic surgery: a mixed-methods study of a diffusion of innovation in healthcare. Healthcare. 2016;4(3):181–7. 27637824 10.1016/j.hjdsi.2015.09.003

