# Supplementary material for: Knowledge translation in surgery: a scoping review of implementation strategies, effectiveness and contextual barriers and enablers
Source: BMC Health Serv Res. 2025 Dec 5;26:48. doi: 10.1186/s12913-025-13369-2 (PMC12797646; doi:10.1186/s12913-025-13369-2)
Supplement: Supplementary file 1 — Supplementary Material 1 [file 12913_2025_13369_MOESM1_ESM.docx]

**SUPPLEMENTARY INFORMATION**

**S1: Search Strategy**

| Review Question 1 | Surgeons[Mesh] OR “Practice Patterns, Physicians” [Mesh] OR “Surgical Procedures, Operative”[Mesh] OR “surgeon* behavior”[tw]  “Implementation Science”[Mesh] OR “Diffusion of Innovation”[Mesh] OR “implement* strateg*”[tw]  "Guideline adherence"[Mesh] OR “Evidence-Based Practice”[Mesh] OR "Translational Research, Biomedical"[Mesh] OR "behavior change"[tw] OR guideline [tw] OR adoption[tw]  "Medical Audit"[Mesh] or “audit and feedback”[tw]  “Education, Medical, Continuing"[Mesh] OR symposi*[tw] OR webinar*[tw]  "Reminder Systems"[Mesh] OR “Prompt*”[tw] OR “reminder*”[tw]  "Education, Medical"[Mesh:NoExp] AND “social media”[tw] |
| --- | --- |
| Review Question 2 | Surgeons[Mesh] OR “Practice Patterns, Physicians” [Mesh] OR “Surgical Procedures, Operative”[Mesh] OR “surgeon* behavior”[tw]  “Implementation Science”[Mesh] OR “Diffusion of Innovation”[Mesh] OR “implement* strateg*”[tw]  "Guideline adherence"[Mesh] OR “Evidence-Based Practice”[Mesh] OR "Translational Research, Biomedical"[Mesh] OR "behavior change"[tw]  "Attitude of Health Personnel"[Mesh:NoExp] OR barrier[tw] OR facilitator [tw] |

**S2: Example of Search String**

| **Date** | November 21, 2024 | |
| --- | --- | --- |
| **Database** | PubMed | |
|  | **Search String** | **Yield** |
|  | ((Surgeons[Mesh] OR "Practice Patterns, Physicians" [Mesh] OR "Surgical Procedures, Operative"[Mesh] OR "surgeon* behavior"[tw]) AND ("Implementation Science"[Mesh] OR "Diffusion of Innovation"[Mesh] OR "implement* strateg*"[tw])) AND ("Guideline adherence"[Mesh] OR "Evidence-Based Practice"[Mesh] OR "Translational Research, Biomedical"[Mesh] OR "behavior change"[tw])**Filters:**in the last 10 years | **153** |
|  | (((Surgeons[Mesh] OR "Practice Patterns, Physicians" [Mesh] OR "Surgical Procedures, Operative"[Mesh] OR "surgeon* behavior"[tw]) AND ("Implementation Science"[Mesh] OR "Diffusion of Innovation"[Mesh] OR "implement* strateg*"[tw])) AND ("Guideline adherence"[Mesh] OR "Evidence-Based Practice"[Mesh] OR "Translational Research, Biomedical"[Mesh] OR "behavior change"[tw]) AND (y_10[Filter])) ("Medical Audit"[Mesh] or "audit and feedback"[tw] AND (y_10[Filter])) Filters: in the last 10 years | **10** |
|  | (((Surgeons[Mesh] OR "Practice Patterns, Physicians" [Mesh] OR "Surgical Procedures, Operative"[Mesh] OR "surgeon* behavior"[tw]) AND ("Implementation Science"[Mesh] OR "Diffusion of Innovation"[Mesh] OR "implement* strateg*"[tw])) AND ("Guideline adherence"[Mesh] OR "Evidence-Based Practice"[Mesh] OR "Translational Research, Biomedical"[Mesh] OR "behavior change"[tw]) AND (y_10[Filter])) AND ("Reminder Systems"[Mesh] OR "Prompt*"[tw] OR "reminder*"[tw] AND (y_10[Filter])) Filters: in the last 10 years | **5** |
|  | (((Surgeons[Mesh] OR "Practice Patterns, Physicians" [Mesh] OR "Surgical Procedures, Operative"[Mesh] OR "surgeon* behavior"[tw]) AND ("Implementation Science"[Mesh] OR "Diffusion of Innovation"[Mesh] OR "implement* strateg*"[tw])) AND ("Guideline adherence"[Mesh] OR "Evidence-Based Practice"[Mesh] OR "Translational Research, Biomedical"[Mesh] OR "behavior change"[tw]) AND (y_10[Filter])) AND ("Education, Medical, Continuing"[Mesh]" OR symposi*[tw] OR webinar*[tw] AND (y_10[Filter])) Filters: in the last 10 years | **2** |
|  |  | **170** |

**S3 – PRISMA flow diagram**

Records identified from*:

Databases (n =970)

Rapid Initial Search (n = 10)

Records removed *before screening*:

Duplicate records removed (n =131)

Records removed for other reasons (n =1) (retracted)

Records identified from:

Related article search & citation searching (n = 18)

**Identification**

Records removed *before screening*:

Duplicate records removed (n =3)

Records screened (Abstract and Title Only)

(n = 848)

Erroneous articles outside scope of study (n=630)

Records screened for Selection Criteria

(n =218)

Reports excluded for not meeting selection criteria

(n=143)

Reports assessed for eligibility

(n = 76)

Decision to include for full text screening by 2 reviewers (n=67)

Decision to review in full text screening by 1 reviewer (n=9)

Reports excluded:

Wrong population (not focused on surgeons) (n = 2)

Wrong outcome (focused on education, training (n = 4)

Wrong intervention (research vs practice) (n = 1)

Reports assessed for eligibility, including study design

(n= 13)

(n = )

Reports excluded:

Study design (n=4)

Not within geographic focus (n = 1)

Wrong population (not focused on surgeons) (n = 2)

Wrong intervention (n=2)

**Screening**

Reports not retrieved

(n = 2)

Reports assessed for eligibility (experimental and quasi-experimental)

(n = 69)

Reports excluded (n=37)

Records screened (Abstract and Title Only)

(n = 15)

Reports included (n=4)

Studies included in review

(N=34)
(n=30 from formal search;
 n=4 from secondary search)

**Included**
